# Perceived Facilitators and Barriers Inform Young Adult Cancer Survivors’ Preferences for Physical Activity and Diet Interventions: A Qualitative Study

**DOI:** 10.3390/nu18172896

**Published:** 2026-09-03

**Authors:** Rashida K. Jones, Aasha I. Hoogland, Bihe Hu, Xiaoyin Li, Carley Geiss, Melinda L. Maconi, Rebecca Blackwell, Yvelise Rodriguez, Crystal Bryant, Nathaly Irizarry-Arroyo, Laura B. Oswald, Brian D. Gonzalez, Brent J. Small, Andrew Galligan, Heather S. L. Jim, Marilyn Stern, Sylvia L. Crowder

**Affiliations:** 1 Department of Health Outcomes and Behavior, H. Lee Moffitt Cancer Center, Tampa, FL 33612, USA; rashida.jones@moffitt.org (R.K.J.);; 2 Participant Research, Interventions, and Measurement (PRISM) Core, H. Lee Moffitt Cancer Center, Tampa, FL 33612, USArebeccablackwellmartinez@gmail.com (R.B.); 3 School of Nursing, University of North Carolina at Chapel Hill, Chapel Hill, NC 27599, USA; 4 Department of Pediatrics, University of South Florida Health Morsani College of Medicine, Tampa, FL 33606, USA; 5 College of Behavioral and Community Sciences, University of South Florida, Tampa, FL 33612, USA

**Keywords:** barriers, cancer survivorship, diet, facilitators, physical activity, young adult cancer

## Abstract

**Purpose:** Young adult cancer survivors aged 20–30 years are at increased risk for chronic health conditions, influenced by modifiable lifestyle behaviors. Many do not meet the American Cancer Society’s guidelines for physical activity and healthy eating. Guided by Social Cognitive Theory, this qualitative study explored the facilitators and barriers to healthy lifestyle behaviors among a racially and ethnically diverse sample of young adult cancer survivors aged 20–30 years to inform tailored interventions. **Methods:** Participants were recruited through the Adolescent and Young Adult Oncology Program at Moffitt Cancer Center in Florida, USA. Semi-structured interviews were conducted and analyzed using applied thematic analysis. Transcripts were coded in NVivo 12 Plus and organized into themes reflecting Social Cognitive Theory constructs and intervention preferences. Demographic information was collected via pre-interview surveys. **Results:** Participants (N = 30) were an average of 26 years (SD = 2.4). Most identified as non-Hispanic (60%), White (60%), unmarried (83%), women (83%), and with annual household income ≥$40,000 (67%). The most common diagnoses were thyroid cancer (20%), lymphoid (17%), and sarcoma (13%). Interviews lasted 30–88 min (M = 52). Five themes emerged: (1) competing demands and time constraints; (2) symptom burden and desire for credible, flexible support; (3) previous habits, self-efficacy, and community building; (4) dual role of social support; and (5) cancer as a catalyst for behavior change. **Conclusions:** Findings identified facilitators and barriers to physical activity and healthy eating, and intervention preferences among young adult cancer survivors, which can inform the timing, content, and modality of tailored interventions. **Implications for Cancer Survivors:** Findings can inform tailored, survivor-centered interventions for young adult cancer survivors.

## 1. Introduction

Compared with peers without a history of cancer, adolescent and young adult cancer survivors (ages 15–39) have a twofold greater risk of developing comorbid conditions driven by the lasting effects of cancer and its treatments [1]. In a retrospective cohort of 6778 adolescent and young adult survivors, 40% reported having multiple comorbidities (≥2) within 12 years of diagnosis, compared with 20% of adolescent and young adults without a cancer history [2]. Common comorbidities include cardiovascular, metabolic, and respiratory conditions [1,2], many of which are strongly influenced by modifiable lifestyle behaviors. These lifestyle-related comorbidities not only contribute to long-term morbidity but also increase the risk of primary and secondary cancers later in life [3,4,5,6].

The American Cancer Society (ACS) provides evidence-based guidelines for cancer survivors to promote long-term health [7]. Consistent with ACS recommendations, adolescent and young adult cancer survivors are encouraged to engage in regular physical activity (e.g., adults: 150–300 min of moderate intensity; adolescents and teens: 60 min of moderate or vigorous intensity per day) and follow a healthy dietary pattern (e.g., a variety of fruits and vegetables, whole grains, with limited red and processed meats, and highly processed foods) [7]. However, adherence to these recommendations remains low among adolescent and young adult cancer survivors [8,9,10]. Nearly one-quarter are classified as inactive after treatment, defined as reporting fewer than 27 metabolic equivalents per week of leisure-time physical activity [8], and approximately three-quarters report inadequate fruit and vegetable intake (≥400 g/day) [9,10]. Low guideline adherence may reflect persistent cancer- and treatment-related effects, such as chronic fatigue [11], as well as psychosocial factors [12], including depression, anxiety, social isolation [13], and impairments in physical mobility [14]. Despite this, the extent to which these physical and psychosocial factors influence adherence to recommended lifestyle behaviors among adolescent and young adult cancer survivors remains poorly understood. Identifying these factors would help inform interventions to improve survivorship outcomes.

Existing studies tend to focus on physical activity [15,16,17,18] or diet [19,20,21,22] in isolation, rather than addressing both in combination, despite their joint importance in the ACS survivorship guidelines [23,24]. Moreover, heterogeneity in study populations and methodologies, including differences in intervention length, type, theoretical frameworks, as well as limited alignment with ACS guidelines, reduces comparability across studies and hinders the identification of effective strategies [25]. Many studies include individuals aged 15–39 years [26,27], spanning various developmental stages and those in active treatment, with limited focus on survivors aged 20–30 years [28,29], who face unique developmental transitions [27,30]. These gaps reveal a lack of integrated, guideline-aligned diet and physical activity interventions tailored for young adult survivors aged 20–30 years.

Guided by Social Cognitive Theory, the aim of this study was to explore perceived facilitators, barriers, and intervention preferences related to physical activity and healthy eating among young adult cancer survivors aged 20–30 years following completion of adjuvant treatment. Findings will inform the development of survivor-focused strategies that are practical, aligned with guideline-concordant care, and effective in encouraging adherence to healthy lifestyle behaviors within this group.

## 2. Methods

### 2.1. Ethical Approval

This study was performed in accordance with the ethical standards of the Declaration of Helsinki and was approved by the Advarra Institutional Review Board (#00000971). All participants were informed of the purpose and procedures of the study and were informed that study participation was voluntary. All participants provided written informed consent before data collection. All data were de-identified, and each participant was assigned an anonymized record ID number. No individuals are identifiable in any presentation of results.

### 2.2. Study Design

This qualitative study was conducted with a subset of participants enrolled in the Individualized **CO**aching in young **A**dult **C**ancer survivors to encourage **H**ealthy behaviors (COACH) study, an observational study in which young adult cancer survivors completed a survey of their current health behaviors, physical activity, and dietary intervention preferences [31]. Individual, semi-structured interviews were conducted to further explore survivors’ intervention preferences previously identified in the parent study.

### 2.3. Participant Recruitment

Young adult cancer survivors were recruited through the Adolescent and Young Adult Program at Moffitt Cancer Center to participate in a larger study on health behaviors in young adults with cancer [i.e., the Individualized **CO**aching in young **A**dult **C**ancer survivors to encourage **H**ealthy behaviors (COACH) study] [31]. Survivors were eligible to participate in the larger study if they were: (1) diagnosed with cancer between the ages of 19 and 29 years old; (2) currently between the ages of 20 and 30 years old; (3) at least one year post-adjuvant treatment; (4) able to read/speak English; (5) able to complete an online survey; and (6) able to consume food orally. Exclusion criteria included women who were pregnant, as altered eating patterns may not represent their typical pre-pregnancy diet.

Participants were recruited from May 2022 to April 2024. Trained research coordinators screened electronic medical records to determine eligibility, then contacted survivors by mailers, phone, text message, e-mail, or in person in-clinic to introduce them to the study, allow time for questions, determine interest in participating, and solicit consent. Young adult cancer survivors who provided contact information were invited to participate in interviews upon completion of the parent study [31]. Upon confirmation of interest, participants completed a consent form via REDCap version 17.3.0 (Research Electronic Data Capture; Vanderbilt University Medical Center, Nashville, TN, USA).

### 2.4. Measures

Participants completed survey measures capturing their health history, including questions on age at diagnosis, time since treatment completion, and sociodemographic characteristics such as age, gender, race, ethnicity, education, income, employment, marital status, smoking status, alcohol, and cannabis use. Clinical information was extracted from medical records and included cancer type, treatment modalities, and the most recent body mass index (BMI).

### 2.5. Data Collection

Semi-structured interviews were conducted by PhD-level qualitative researchers (MM and RB) via teleconference (i.e., ZOOM version 7.0.0). Interviewers were trained social scientists with no prior relationship to participants. Interview questions explored perceived facilitators and barriers to physical activity and healthy eating, followed by a discussion of preferred formats and topics for future interventions for young adult survivors. After the interview, participants received a $35 gift card. Survey data were collected and managed using REDCap, an electronic data capture tool hosted at H. Lee Moffitt Cancer Center [32,33]. Medical record data were collected and abstracted from PowerChart, version 2018.01 (Oracle Health, Austin, TX, USA).

A total of 33 individuals from the parent study were approached; all agreed to participate and were scheduled for interviews. Three interviews were not conducted due to loss to follow-up (*n* = 2) and participant withdrawal (*n* = 1). A total of 30 semi-structured interviews were conducted, ranging in length from 30 to 88 min (Mean = 52). The interview guide is provided in Supplemental File S1. Interviews continued until saturation was reached (i.e., no new themes were identified).

### 2.6. Data Analysis

All interviews were transcribed verbatim by a third-party (GMR Transcription), reviewed for accuracy by interviewers, de-identified, and analyzed following the tenets of applied thematic analysis [34]. An initial codebook was developed based on the semi-structured interview guide, emerging themes identified during data collection, and regular study team meetings. MM and RB conducted intercoder reliability checks and codebook refinement. An acceptable level of intercoder reliability was reached (Cohen’s Kappa = 0.82) after three rounds of independent coding and comparison [35]. Discrepancies were resolved through consensus. Transcripts were then coded using NVivo 12 Plus software, version 12.0 (QSR International Pty Ltd.: Melbourne, VIC, Australia) [36] and organized into primary and secondary themes related to the socio-cognitive factors that impact young adult survivors’ dietary and physical activity behaviors, as well as perceptions of proposed interventions [35,37]. Participant characteristics were described using means, standard deviations, frequencies, and percentages. All statistical analyses were performed using SAS, version 9.4 (Cary, NC, USA) [38].

## 3. Results

### 3.1. Participant Characteristics

Demographic and clinical characteristics are presented in Table 1. Participants had a mean age at survey of 26 years (SD = 2.4). Most identified as non-Hispanic (60%), White (60%), unmarried (83%), women (83%), and with an annual household income ≥$40,000 (67%). The most common diagnoses were thyroid cancer (20%), lymphoid cancer (17%), and sarcoma (13%). All participants were at least one-year post-adjuvant treatment completion (range 1–4 years).

### 3.2. Summary of Themes

Thematic analysis yielded five overarching themes related to participants’ experiences, perceived facilitators and barriers to physical activity and healthy eating, and preferences for lifestyle interventions. Themes included: (1) competing demands and time constraints; (2) persistent symptom burden and the desire for credible, flexible support; (3) previous habits, self-efficacy, and community building; (4) dual role of social support; and (5) cancer as a catalyst for behavior change. Participant quotes are identified by participant number, age, cancer staging, and cancer type. A detailed description of the themes and illustrative quotes is presented in Table 2.

### 3.3. Competing Demands and Time Constraints

Participants consistently described how work, school, and family responsibilities affected their capacity to maintain a healthy lifestyle, including limited time to engage in regular physical activity and prepare and consume balanced meals. Fast food was viewed as quick, affordable, and more convenient than preparing a meal. As one participant explained, “It’s just so easy to go to places like that … especially after work. After a long day, I don’t want to sit and cook” (P57, 22-year-old Stage I thyroid cancer survivor). Even when participants recognized healthier options, convenience would outweigh those intentions. One participant noted, “There is healthier ways to do it. And I know that. It’s just the ease and the comfort of being like, “I don’t want to get off the couch right now ‘cause I just went to the gym. And I’m gonna order whatever I want” (P55, 29-year-old leukemia survivor, unknown stage).

Parenting responsibilities further constrained structured physical activity, although some participants adapted their routines to incorporate exercise during time spent with children. For example, one participant shared, “We’re swimming. We’re doing a lot of activities together. But other than that, I haven’t went [to the gym] in a while” (P03, 27-year-old, Stage III lymphoid cancer survivor). Other parents mentioned that their children served as a source of motivation to model healthy behaviors.

In addition to time scarcity, participants frequently cited the high cost of healthy foods (e.g., fruits and vegetables) as an obstacle to following a healthy diet aligned with the ACS’s diet and physical activity recommendations for cancer prevention and management. Similarly, participants described the cost-prohibitive nature of wellness programs. As one participant noted, “If it’s free, I would absolutely participate. With a little guidance, I could do more physically” (P12, 25-year-old Stage 0 [other cancer type] survivor), highlighting how financial accessibility may facilitate engagement in future physical activity or healthy eating interventions. Many participants were transitioning into careers or graduate school programs and reported prioritizing occupational or academic demands over physical activity and healthy eating.

### 3.4. Persistent Symptom Burden and the Desire for Credible, Flexible Support

Participants generally regarded physical activity as “very important,” yet many struggled to return to pre-cancer activity levels due to lingering treatment effects. Fatigue was among the most frequently cited symptoms of cancer treatment that continued into survivorship, often limiting participants’ ability to perform daily tasks. Others described more severe or long-lasting impairments, including reduced strength, mobility limitations, or new comorbidities following treatment that hindered engagement in regular physical activity. Two years after completing chemotherapy and surgery for brain cancer, one participant shared: “I was blind through the corner of my eyes. I was paralyzed on the left side of my body. I’m still regaining function. As of right now, I still feel tingling on my left side of my body. I take vitamins and go to the gym to help with that. I was also diagnosed with MS [multiple sclerosis] after my brain surgery” (P31, 29-year-old brain cancer survivor, unknown stage).

Rapid weight fluctuations affected participants’ motivation and confidence in their physical abilities. Some experienced increased emotional distress, which decreased their willingness to engage in physical activities in survivorship. One participant noted, “Things have changed though like I gained weight. I don’t feel like I want to go through the effort of wanting to do exercise” (P32, 28-year-old Stage I sarcoma survivor). However, for some participants, weight management was not the primary motivator; they emphasized adopting gradual, sustainable health behaviors rather than focusing on weight. As described by a hematologic cancer survivor, “I’ve been trying to make healthier choices rather than to like calorie count or restrict anything… I would like to lose weight but it’s a little hard so I’m just trying to make it slow and not—like more so healthy habits than trying to just make it about losing weight” (P13, 27-year-old lymphoid cancer survivor, unknown stage).

Similarly, participants generally associated dietary intake with health outcomes. Although good nutrition (e.g., high consumption of fruits and vegetables, frequent water intake, low consumption of sugar, sodium, processed foods, and unhealthy fats) was viewed as “very important,” many participants reported difficulty meeting their nutritional goals. Treatment-related symptoms, including nausea, taste changes, and reduced appetite, made maintaining a balanced diet challenging during and after treatment.

In the absence of structured guidance, participants often relied on online resources for nutrition and physical activity recommendations, including educational websites and social media influencers. While participants preferred easy access and free resources, many described challenges in identifying credible sources. One participant noted, “I would talk to my gastro[enterologist] and he would offer a little bit. But I would have to do so much research on my own on just Google. And it’s really hard to find certified sources and stuff that’s not just biased or personal experience. And just the right information” (P47, 27-year-old, Stage I thyroid cancer survivor).

Given these challenges, participants expressed interest in structured guidance, such as access to qualified professionals to ensure exercises were appropriate and performed correctly. Additionally, participants repeatedly emphasized the need for trustworthy, accurate, and consistent nutrition information, reflecting their experiences with conflicting advice from physicians and dietitians during treatment. Some highlighted the importance of integrating nutrition guidance into cancer care so patients aren’t left to navigate information independently and can rely on recommendations tailored specifically to cancer contexts. This included a desire for practical education, including “learning how to read the labels…I think that would be amazing” (P03, 27-year-old Stage III lymphoid cancer survivor). At the same time, participants described confusion arising from inconsistent messaging across providers, noting that recommendations often varied widely, even in simple cases such as whether to avoid common foods like pizza and supplements such as probiotics, leaving them uncertain about what advice to follow. Overall, participants underscored the need for more comprehensive, personalized support beyond receiving “a piece of paper”.

### 3.5. Previous Habits, Self-Efficacy, and Community Building

#### 3.5.1. Previous Habits as a Motivator

Previous healthy habits emerged as a key facilitator of physical activity and healthy eating. Some participants who engaged in healthy behaviors before their cancer diagnosis reported fewer challenges in returning to these habits during survivorship. As one survivor explained, “Well, prior to cancer, I was always quite active and always ate quite healthy. And then after cancer, I’ve just been a little bit more conscious of my fruits and vegetable intake” (P72, 26-year-old Stage II breast cancer survivor).

However, previous habits could also serve as a source of frustration. Participants who had been highly active before their cancer diagnosis often compared their current physical abilities to their pre-diagnosis baseline and expressed disappointment when treatment-related changes limited their ability to engage in physical activity with the same intensity or frequency. For some survivors, these challenges reduced motivation to be active, as one participant explained, “I go to the gym once in a while… I’m not as dedicated as I was before… It’s so frustrating in my mind that I was at this point. And now I’m not even 10 steps back…I’m like 10 miles back from where I was” (P55, 29-year-old leukemia survivor, unknown stage). For others, prior activity levels were both a motivator to regain former habits and a reminder of capabilities that had changed following treatment. One survivor reflected, “I feel as though my left side is not as strong as what it used to be… I would get into a mental space where this used to be easy for me. Why is this so hard? And then I have to remember my body’s not what it used to be and it’s okay” (P72, 26-year-old Stage II breast cancer survivor).

Similarly, those who found physical activity (e.g., running, strength training, cycling) enjoyable and liked the taste of nutrient-dense foods (e.g., lean proteins, fruits, vegetables) before their diagnosis described greater ease in maintaining these behaviors into survivorship. Environmental factors also played a role; Florida’s warm weather and scenic outdoor settings were frequently cited as motivators. One participant described a monthly outdoor yoga class at a nearby pier as, “such a beautiful setting outside, and the setting is just perfect, and it really sets the mood for the whole class and all the people that attend” (P09, 24-year-old Stage II lymphoid cancer survivor).

#### 3.5.2. Self-Efficacy as a Tool for Adopting and Maintaining Healthy Habits

Participants without established healthy routines before their cancer diagnosis often reported continued difficulty making dietary changes. One participant shared, “I have always eaten junk food…I never found it easy to cook a meal” (P32, 28-year-old Stage I sarcoma survivor). To support healthy eating, some participants described intentionally enhancing the appeal of healthy foods by using preferred condiments or seasonings.

For physical activity, many reported that team sports or group-based activities increased self-efficacy. One participant explained, “what helps the most is doing a sport. … It could be flag football, or it could be kickball, or it could be tennis—anything that kinda gets me up and going—that helps too” (P14, 27-year-old Stage II thyroid cancer survivor).

#### 3.5.3. Peer Connection as a Facilitator of Engagement

Participants expressed a strong interest in connecting with other young adult cancer survivors, with many favoring group formats. One participant mentioned that joining an intervention would be appealing “mostly because of the community support aspect of it… with people who are going through something similar” (P14, 27-year-old Stage II thyroid cancer survivor). Regarding group-based physical activity intervention, participants preferred group fitness classes that incorporated fun, creative elements such as kickboxing, strength training, or dance. For group-dietary intervention, participants expressed interest in cooking demonstrations to make nutritious meals more appealing and to increase their nutritional knowledge. While opinions varied on who should lead future interventions, most agreed a physical therapist or personal trainer with similar experience should guide the physical activity component and a nutritionist or dietitian experienced in cancer care should oversee the dietary component. Some also highlighted the potential benefits of survivor-led or co-led sessions, believing that those with lived experience could offer more relatable guidance:

“I would like to see somebody’s who’s actually been diagnosed to really talk about that, because sometimes these doctors are so in a different planet. They understand the disease, but they don’t understand the side effects. They don’t understand the mental, all of the loads that these cancer patients carry to be able to even think about cooking and recipes and all of that. So, sometimes I feel like when you actually go through it and you’re on the other side of it, I think can relate a bit better”.

(P03, 27-year-old Stage III lymphoid cancer survivor)

### 3.6. Dual Role of Social Support

Participants frequently viewed their social networks as vital for maintaining healthy behaviors. During treatment, friends and family offered practical assistance, like preparing nutritious meals, which helped participants cope with fatigue or emotional stress related to their cancer diagnosis. After treatment, ongoing encouragement from family and peers continued to influence eating habits. One participant shared how changes in her social circle had a positive impact on her diet stating, “a lot of my friends are extremely health conscious lately… but definitely my parents who eat super healthy and a lot of my girlfriends who eat extremely healthy …influence a lot of my diet” (P55, 29-year-old leukemia survivor, unknown stage).

Social support also facilitated physical activity through companionship or informal guidance. As one participant reflected, “my sister… started going to the gym; so then, I wanted to” (P12, 25-year-old Stage 0, [other cancer type] survivor). However, social support was not uniformly beneficial. Some participants described tension when family members provided foods high in sugar or unhealthy fats that were inconsistent with their health goals. As one survivor noted, “I get healthy [meals] from one side of the family and not-so-healthy from the other” (P55, 29-year-old leukemia survivor, unknown stage). Additionally, when dietary recommendations for cancer survivors conflicted with cultural food preferences, participants struggled to find alternatives, resist comfort-food cravings, or politely decline help, leading to disappointment and frustration. This frustration is illustrated by one participant, who explained: “I’m sad because my mom is Honduran and my dad is Lebanese… but they use a lot of garlic and a lot of onion, and I can’t have either anymore… and a lot of tomato also can’t eat” (P47, 27-year-old, Stage I thyroid cancer survivor).

Importantly, these challenges frequently overlap with broader feelings of social disconnection. While family and friends were viewed as sources of emotional support, some participants reported feeling intensely isolated, and many described feeling disappointed by their support systems, mentioning the end of romantic relationships and close friendships after diagnosis. One participant shared:

“I lost a really close friend. They did not want to [be] in my life when I had got diagnosed with cancer…. I think [you lose] friends because you are just going through your situation, having anxiety and dealing with not wanting to be in certain settings or because you’re forgetful, being scared …sometimes [you] go out alone”.

(P31, 29-year-old brain cancer survivor, unknown stage)

Even among those with otherwise strong support networks, many reported that despite good intentions, family and friends could not relate to their experience. Some participants distanced themselves or rejected sympathy or support from others, citing concerns about being a burden, or not feeling sick enough to warrant assistance, which contributed to experiences of survivor’s guilt. This emotional incongruence is reflected in one participant’s reflection:

“Survivor’s guilt …was the biggest emotional issue that I faced because it was like…the good cancer, the cancer that you want… So, it’s like, well, I shouldn’t be having these feelings. I shouldn’t be sad. I shouldn’t be crying randomly… I’m too afraid to talk about them or express them because I had the good cancer, and my prognosis was good”.

(P14, 27-year-old Stage II thyroid cancer survivor)

### 3.7. Cancer as a Catalyst for Behavior Change

For many participants, receiving a cancer diagnosis encouraged them to reflect on their health behaviors and long-term risks. Several expressed greater awareness of their mortality, which motivated lifestyle changes, highlighting the role of physical activity and healthy eating in recovery, lowering recurrence risk, and preventing further health issues. One participant described this shift as a “little bit of a reality check,” explaining that she changed her lifestyle, becoming “very, very active” and paid more attention to nutrition labels (P43, 28- year-old Stage II thyroid cancer survivor).

Participants expressed diverse preferences regarding when to receive interventions. Some preferred to learn about physical activity and healthy eating in combination at the time of diagnosis, allowing them to initiate lifestyle changes immediately. As one participant noted, “I think that immediately. Looking back, some things with my care I wish were more immediate. I wish I would’ve start doing” (P31, 29-year-old brain cancer survivor, unknown stage). In contrast, others favored beginning nutrition programs during treatment to help manage side effects and establish healthy habits. One participant explained, “I think during all of it. When I was doing chemo, I wasn’t sure if the food I was eating was good. I think throughout the whole process from beginning to end and after” (P42, 28-year-old Stage II breast cancer survivor).

Preferences for the timing of physical activity also varied. Many participants favored initiating physical activity during or immediately after treatment. Others, however, preferred delaying activity until one to six months post-treatment or later, depending on their recovery, readiness, and personal circumstances. This perspective was illustrated by one participant who stated, “Probably at least six months after treatment…I think I had to wait until like—I think I did wait like six months after my surgery and everything. So, maybe like six months, so then you give your body time to heal, and rest, and recover before starting a physical workout routine” (P12, 25-year-old Stage 0, [other cancer type] survivor).

## 4. Discussion

This qualitative study examined facilitators and barriers to physical activity and healthy eating, and preferences for physical activity and dietary interventions. Several themes emerged: individual factors affecting physical activity and diet; interpersonal desires for peer support and community; and institutional considerations related to access to credible information on healthy lifestyles. Additional themes reflected sociocultural aspects unique to young adults, such as limited nutritional knowledge, low self-efficacy, and demands related to early career or post-secondary education.

### 4.1. Facilitators

Some participants described their cancer diagnosis as a catalyst for increasing physical activity and improving diet. These findings align with a prior study of 33 healthcare workers (e.g., dietitians, nurses, oncologists, primary care providers, social workers, patient navigators, survivorship coordinators) [39]. In this qualitative study, healthcare workers perceived a younger age at cancer diagnosis as a key facilitator of behavior change, noting that younger patients were more likely to adopt healthier lifestyle changes due to greater concern about mortality [39]. This heightened awareness of health risks may suggest that educational interventions that accurately communicate risk may be particularly motivating for young adult survivors.

Participants also sought information online and on social media, with many following fitness and nutrition influencers for guidance, knowledge, and motivation for physical activity and healthy eating. The high level of internet engagement in this population highlights an important consideration for intervention development, suggesting that incorporating digital and social media components may improve engagement among young adult cancer survivors [15,16]. Positive social support from family and friends facilitates the adoption of healthy behaviors. Relationships that provided tangible assistance or emotional encouragement were generally viewed as supportive and helped participants engage in physical activity and healthy eating. These findings appear consistent with the literature, which frequently identifies family as an important source of support for young adult cancer survivors and underscores the nuanced role that social relationships play in shaping health behaviors [40,41,42]. For example, Wickramasinghe, Fern, Taylor and Feltbower [41] demonstrated that support from family members and partners was positively associated with greater perceived support from friends, suggesting that social support across relationship domains is clustered, with family, friends, and partners influencing one another. Together, these findings highlight the intricate and linked aspects of social support and relationships.

### 4.2. Barriers

Despite its potential to facilitate behavior change, social support also acted as a barrier when family members or peers were not aligned with participants’ health goals. Participants described instances in which social environments discouraged physical activity or healthy eating, or in which their social networks failed to adopt similar lifestyle changes. In some cases, these differences contributed to feelings of isolation, particularly when survivors perceived a lack of shared experiences or understanding during their cancer journey. Consistent with prior research, such disconnects within social networks have been shown to serve as barriers to engagement in healthy lifestyle behaviors [43].

Survivors in our study described challenges connecting with loved ones who were unable to empathize with the invisible effects of cancer (e.g., anxiety, memory problems, fatigue), leading to social distancing, avoidant behaviors, and, in some cases, the loss of close relationships over time. A recent mixed-methods study of 175 adolescent and young adult cancer survivors similarly highlighted how differences in experiences and understanding between survivors and their social networks contributed to social disconnection [44]. Collectively, these findings highlight the complex and multifaceted role of interpersonal relationships during cancer treatment and survivorship.

Chronic fatigue was a salient barrier to physical activity and healthy eating, reported by nearly half of participants (*n* = 13). Notably, when interviewed, participants were, on average, about three years post-cancer treatment, indicating that fatigue persisted well beyond the active treatment period. Previous research has consistently shown that cancer-related fatigue is a substantial problem among young adult cancer survivors and is negatively associated with healthy lifestyle behaviors and quality of life [11,43,45,46,47]. Additionally, participants described competing demands during treatment and survivorship, including work, school, and family responsibilities. These findings align with previous research suggesting that young adult survivors face persistent fatigue and psychosocial challenges that can interfere with daily functioning and quality of life, while also navigating age-specific responsibilities such as education, career trajectories, and family roles during a critical developmental period [46,47,48].

Compounding these challenges, the socioeconomic impact of cancer on young adults is substantial. Compared with older adult cancer survivors and age-matched peers without cancer, young adult survivors are more likely to experience financial hardship because of their cancer diagnosis [49,50,51]. Because most wage growth occurs in the first decade of a career, disruptions from cancer treatment and treatment-related symptoms may lead to greater financial hardship in later life [51,52]. When combined, persistent fatigue, competing demands, and financial strain may limit young adult survivors’ capacity to maintain physical activity and healthy eating behaviors over the long term, underscoring the need for flexible, low-cost, and supportive interventions tailored to their unique needs and life stage.

### 4.3. Intervention Preferences

The intervention preferences expressed by participants closely reflected the facilitators and barriers identified during thematic analysis and informed several key components of the proposed intervention (Table 3). Young adult survivors in this study consistently highlighted a strong wish to connect with other young adult cancer survivors to foster community and social support. Participants preferred group physical activities that accommodate various physical abilities and provide skills essential to recovery, such as strength training and cardiovascular conditioning. They also preferred group-based dietary programs with cooking demonstrations. Collectively, their experiences emphasize the need for a developmentally appropriate, survivor-focused program that fosters community and accountability, while addressing the time limitations, limited resources, and emotional challenges faced by young adult survivors. Some participants also noted the importance of interventions to be led or co-led by fellow survivors, highlighting the importance of peer-driven, relatable guidance. Consistent with these findings, prior research has shown that social isolation is common among young adult cancer survivors and is associated with increased psychological distress, while social connectedness may serve as a mechanism to improve psychological well-being [13,53]. Strategies to reduce social isolation may therefore focus on strengthening support systems by increasing opportunities for connection with other young adult survivors who share similar experiences [53]. Such approaches may provide meaningful support while also enhancing engagement and interest in physical activity and healthy eating interventions [25,54].

Participants preferred low-cost physical activity and diet interventions, consistent with previous research identifying cost as a barrier to engagement among young adult cancer survivors [55]. Participants also expressed a desire for accurate, relevant nutrition information from qualified professionals (e.g., registered dietitians, exercise specialists, physical therapists), tailored to the needs of young adult survivors. Consistent with these findings, recent young adult survivorship models emphasize the importance of high-quality nutrition and lifestyle counseling delivered by credentialed practitioners, highlighting gaps in current survivorship care that mirror participants’ expressed needs [56]. Accordingly, future interventions should prioritize affordability and incorporate multidisciplinary teams of qualified experts to promote physical activity and healthy eating.

The preferences of the young adult survivors in our study closely align with the constructs of Social Cognitive Theory [37]. Participants reported limited confidence in maintaining healthy lifestyle behaviors, highlighting the importance of self-efficacy, a central determinant of behavior change. Preferences for peer-led and group-based interventions suggest opportunities for observational learning, in which young adult survivors can acquire knowledge, skills, and confidence by observing and interacting with peers who share similar lived experiences. Participants also expressed interest in credible, risk-informed information on the role of healthy eating and physical activity in secondary cancer prevention and recurrence, which reflects heightened awareness that may further motivate engagement in healthy behaviors. Additionally, barriers related to time constraints, financial burden, symptom burden, and social support illustrate the dynamic interplay among individual, environmental, and behavioral factors, consistent with reciprocal determinism. Collectively, these findings suggest that interventions incorporating components at each level may leverage key mechanisms within the Social Cognitive Theory to improve engagement, adherence, and long-term behavior change among young adult cancer survivors.

### 4.4. Alignment and Divergence Between the Parent Trial and Qualitative Findings

While the parent trial and qualitative findings were largely complementary, some differences emerged in modality, delivery, and timing of the interventions. Regarding delivery and modality, participants in the parent trial preferred one-on-one formats, with dietary counseling delivered online and physical activity components delivered in person [31]. In contrast, qualitative participants emphasized a preference for in-person, group-based formats to facilitate peer connection, while still recognizing the convenience of online delivery. This discrepancy may reflect differences in question wording in the interview guide, as well as the use of planned probes that encouraged more detailed responses from participants.

In line with our quantitative findings, most participants emphasized that a registered dietitian should lead the dietary intervention [31]; however, when given the opportunity to co-lead with a cancer patient or survivor, some participants (*n* = 3) viewed this collaboration as particularly beneficial. While this preference was less commonly noted in the parent trial, qualitative participants suggested that survivor involvement could enhance relatability and provide meaningful peer support when integrated with professional expertise. Regarding intervention timing, participants in the parent study favored initiating physical activity and dietary interventions before treatment [31]. In contrast, participants in our study did not reach consensus on an ideal start time but emphasized the importance of being informed about and having access to both physical activity and healthy eating programs at the time of diagnosis.

These findings from both studies have informed the development of the future intervention. Specifically, given participants’ desire for peer connection while valuing convenience, the intervention will use a group-based format with opportunities for peer interaction and flexible delivery approaches to maximize accessibility. Additionally, although participants did not identify a single preferred intervention start time, findings support introducing lifestyle intervention resources at diagnosis and allowing survivors to engage when they feel ready and able to participate.

### 4.5. Strengths and Limitations

This study has several notable strengths. First, conducting in-depth interviews with young adult cancer survivors, a population underrepresented in qualitative research, provides an opportunity to uncover the lived experience of this group and identify facilitators and barriers to physical activity and healthy eating. Importantly, limiting the sample to 20–30 years of age, rather than the commonly used 15–39-year-old range, reduces developmental heterogeneity and strengthens the relevance of the findings for this specific life stage. Next, the work was guided by Social Cognitive Theory, which frames the findings through its constructs of self-efficacy, social support, environmental adaptation, and risk awareness, thereby enhancing the interpretability of the results. Additionally, 40% of our sample identified as Hispanic, a population that is often underrepresented and difficult to recruit in behavioral research. Including the perspectives of Hispanic young adult survivors is particularly important, as culturally informed insights can enhance the relevance, acceptability, and, ultimately, the uptake of future interventions within an ethnically diverse population.

Despite efforts to recruit a racially and gender-diverse sample, study participants were predominantly White, unmarried females, and represented a narrow age range (20–30 years). Although this age range was intentionally selected to reduce developmental heterogeneity, the findings may not transfer to older young adult cancer survivors whose responsibilities and survivorship experiences may differ. In addition, the distribution of cancer diagnoses and treatment modalities within the sample may limit transferability, as survivors with other cancer types or treatment experiences may face different facilitators and barriers to physical activity and healthy eating. As the study was conducted at a single NCI-designated comprehensive cancer center, survivors receiving care in other clinical or community-based settings may have different experiences, resources, and support needs that were not captured. Additionally, individuals with a pre-existing interest in, or heightened need for, physical activity and dietary interventions may have been more likely to participate in the study.

## 5. Conclusions

This study identified key facilitators, barriers, and intervention preferences shaping physical activity and healthy eating preferences among young adult cancer survivors. These findings provide actionable insights to inform the design of developmentally appropriate, guideline-centered, accessible, and survivor-centered interventions. Future research should build on these findings by testing tailored intervention strategies in larger, more diverse samples through randomized clinical trials.

## Figures and Tables

**Table 1:** Demographic and Clinical Characteristics of Young Adult Cancer Survivors N = 30.

Characteristic	Total N (%)
Age at diagnosis: mean ± SD [range], years	22.7 ± 2.2 [21–24]
Age at survey: mean ± SD [range], years	26.3 ± 2.4 [25–28]
Treatment completion: mean ± SD [range], years	2.8 ± 1.6 [1–4]
Female	25 (83)
Non-Hispanic	18 (60)
White	9 (50)
Black or African American	3 (17)
Asian	2 (10)
American Indian or Alaska Native	1 (6)
Other, more than one race	3 (17)
Hispanic	12 (40)
White	9 (75)
Black or African American	1 (8)
Other, more than one race	2 (17)
College Graduate	16 (53)
Annual household income *	
≥$40,000	18 (67)
Marital status **	
Not Married	24 (83)
Employment	
Employed	17 (57)
Body mass index (BMI)	
Healthy weight (BMI 18.5–24.9)	10 (33)
Overweight (BMI 25.0–29.9)	8 (27)
Obese (BMI ≥ 30)	12 (40)
Cancer treatment? ***	
Chemotherapy	22 (73)
Radiation	11 (37)
Immunotherapy	1 (3)
Hormonal	2 (7)
Surgery	22 (73)
Transplant	3 (10)
Tumor site	
Thyroid	6 (20)
Lymphoid	5 (17)
Sarcoma	4 (13)
Testis	2 (7)
Leukemia	3 (10)
Breast	3 (10)
Brain	3 (10)
Other	4 (13)
Current smoker	1 (3)
Ever smoker ****	3 (10)
Cannabis use	
No	18 (60)
Yes, marijuana or THC	8 (26)
Yes, cannabidiol or CBD	2 (7)
Yes, THC and CBD	2 (7)
Alcohol use	
Yes	18 (60)

* *n* = 27, ** *n* = 29, *** possible to answer more than one item per question, and **** *n* = 29.

**Table 2:** Qualitative Description of Themes and Select Illustrative Quotes from Young Adult Cancer Survivors.

Theme	Subtheme	Exemplar Quote
Competing Demands and Time Constraints *Work, family, academic, and financial responsibilities limited participants’ ability to engage in regular physical activity and healthy eating highlighting a need for flexible, low-cost lifestyle interventions.*		“It’s just so easy to go to places like that instead of—especially after work. After a long day, I don’t want to sit and cook” (P57, 22-year-old Stage I Thyroid cancer survivor).
		“There is healthier ways to do it. And I know that. It’s just the ease and the comfort of being like, “I don’t want to get off the couch right now ‘cause I just went to the gym. And I’m gonna order whatever I want” (P55, 29-year-old leukemia survivor, unknown stage).
		“We’re swimming. We’re doing a lot of activities together. But other than that, I haven’t went [to the gym] in a while” (P03, 27-year-old, Stage III lymphoid cancer survivor).
		“If it’s free, I would absolutely participate. With a little guidance, I could do more physically” (P12, 25-year-old Stage 0 [other cancer type] survivor).
Persistent Symptom Burden and the Desire for Credible, Flexible Support *Participants valued healthy behaviors but faced physical and dietary challenges from lasting treatment effects, functional limitations, and uncertainty about credible health information, highlighting a need for personalized professional support*		“I was blind through the corner of my eyes. I was paralyzed on the left side of my body. I’m still regaining function. As of right now, I still feel tingling on my left side of my body. I take vitamins and go to the gym to help with that. I was also diagnosed with MS [multiple sclerosis] after my brain surgery” (P31, 29-year-old brain cancer survivor, unknown stage).
		“Things have changed though like I gained weight. I don’t feel like I want to go through the effort of wanting to do exercise” (P32, 28-year-old Stage I sarcoma survivor).
		“I’ve been trying to make healthier choices rather than to like calorie count or restrict anything… I would like to lose weight but it’s a little hard so I’m just trying to make it slow and not—like more so healthy habits than trying to just make it about losing weight” (P13, 27-year-old lymphoid cancer survivor, unknown stage).
		“I would talk to my gastro[enterologist] and he would offer a little bit. But I would have to do so much research on my own on just Google. And it’s really hard to find certified sources and stuff that’s not just biased or personal experience. And just the right information” (P47, 27-year-old, Stage I thyroid cancer survivor).
Previous Habits, Self-Efficacy, and Community Building *Previous healthy habits, confidence in one’s ability to adopt healthy habits, and opportunities for peer connection facilitated engagement in physical activity and healthy eating.*	Previous Habits as a Motivator	“Well, prior to cancer, I was always quite active and always ate quite healthy. And then after cancer, I’ve just been a little bit more conscious of my fruits and vegetable intake” (P72, 26-year-old Stage II breast cancer survivor).
		“I go to the gym once in a while… I’m not as dedicated as I was before… It’s so frustrating in my mind that I was at this point. And now I’m not even 10 steps back…I’m like 10 miles back from where I was” (P55, 29-year-old leukemia survivor, unknown stage).
		“I feel as though my left side is not as strong as what it used to be… I would get into a mental space where this used to be easy for me. Why is this so hard? And then I have to remember my body’s not what it used to be and it’s okay” (P72, 26-year-old Stage II breast cancer survivor).
		“Once a month, there’s a yoga class at a nearby pier, and it’s such a beautiful setting outside, and the setting is just perfect, and it really sets the mood for the whole class and all the people that attend” (P09, 24-year-old Stage II lymphoid cancer survivor).
	Self-Efficacy and Adopting New Habits	“I have always eaten junk food… I never found it easy to cook a meal” (P32, 28- year-old Stage I sarcoma survivor).
		“What helps the most is doing a sport. So, if it’s…any sport, really. It could be flag football, or it could be kickball, or it could be tennis—anything that kinda gets me up and going—that helps too” (P14, 27-year-old Stage II thyroid cancer survivor).
	Peer Connection and Community Building	“Mostly because of the community support aspect of it… with people who are going through something similar” (P14, 27-year-old Stage II thyroid cancer survivor).
		“I would be the first one to sign up. I would love to have a regular group fitness class with people who’ve gone through similar experiences” (P09, 24-year-old Stage II lymphoid cancer survivor).
		“I would like to see somebody’s who’s actually been diagnosed to really talk about that, because sometimes these doctors are so in a different planet. They understand the disease, but they don’t understand the side effects. They don’t understand the mental, all of the loads that these cancer patients carry to be able to even think about cooking and recipes and all of that. So, sometimes I feel like when you actually go through it and you’re on the other side of it, I think can relate a bit better, but that’s just my opinion” (P03, 27-year-old Stage III lymphoid cancer survivor).
Dual Role of Social Support *Family and peers could both support and hinder healthy lifestyle behaviors through practical assistance, social influence, and cultural considerations*		“A lot of my friends are extremely health conscious lately… but definitely my parents who eat super healthy and a lot of my girlfriends who eat extremely healthy …influence a lot of my diet” (P55, 29-year-old leukemia survivor, unknown stage).
		“My boss, she’d bring me some snacks and stuff. Everybody’s pretty healthy here. So, I think they’re a really good, positive influence on me… I think they help me a lot” (P03, 27-year-old, Stage III lymphoid cancer survivor).
		“My sister… started going to the gym; so then, I wanted to” (P12, 25-year-old Stage 0, [other cancer type] survivor).
		“I get healthy [meals] from one side of the family and not-so-healthy from the other” (P55, 29-year-old leukemia survivor, unknown stage).
		“I’m sad because my mom is Honduran and my dad is Lebanese… but they use a lot of garlic and a lot of onion, and I can’t have either anymore… and a lot of tomato also can’t eat” (P47, 27-year-old, Stage I thyroid cancer survivor).
		“I lost a really close friend. They did not want to [be] in my life when I had got diagnosed with cancer…. I think [you lose] friends because you are just going through your situation, having anxiety and dealing with not wanting to be in certain settings or because you’re forgetful, being scared …sometimes [you] go out alone” (P31, 29-year-old brain cancer survivor, unknown stage).
		“Survivor’s guilt …was the biggest emotional issue that I faced because it was like…the good cancer, the cancer that you want… So, it’s like, well, I shouldn’t be having these feelings. I shouldn’t be sad. I shouldn’t be crying randomly… I’m too afraid to talk about them or express them because I had the good cancer, and my prognosis was good” (P14, 27-year-old Stage II thyroid cancer survivor).
Cancer as a Catalyst for Behavior Change *A cancer diagnosis often increased motivation to adopt healthier lifestyles, although preferences for the timing and delivery of interventions varied based on recovery and readiness.*		“I think that immediately. Looking back, some things with my care I wish were more immediate. I wish I would’ve start doing” (P31, 29-year-old brain cancer survivor, unknown stage).
		“I think during all of it. When I was doing chemo, I wasn’t sure if the food I was eating was good. I think throughout the whole process from beginning to end and after” (P42, 28-year-old Stage II breast cancer survivor).
		“Probably at least six months after treatment…I think I had to wait until like—I think I did wait like six months after my surgery and everything. So, maybe like six months, so then you give your body time to heal, and rest, and recover before starting a physical workout routine” (P12, 25-year-old Stage 0, [other cancer type] survivor).

**Table 3:** Translation of Identified Facilitators and Barriers into Key Components for COACH Intervention Development.

Theme	Key Finding (Facilitator or Barrier)	Implication for Intervention Development
Competing Demands and Time Constraints	Barrier: Time, financial burden, and competing role demands limit healthy lifestyle adherence	Offer low-cost hybrid or virtual delivery formats to help reduce structural and logistical barriers to participation
Persistent Symptom Burden and the Desire for Credible, Flexible Support	Barrier: Ongoing treatment and cancer-related symptoms interfere with physical activity and healthy eating	Provide accurate, relevant information from qualified professionals (e.g., registered dietitians, exercise specialists, physical therapists), tailored to the needs of young adult survivors
	Barrier: Difficulty identifying credible health information	
Previous Habits, Self-Efficacy, and Community Building	Barrier: Limited confidence in ability to maintain healthy behaviors	Include peer support and group-based interventions that foster confidence, are engaging, and enhance skill-building
	Barrier: Lack of connection with peers who share similar experiences	
Dual Role of Social Support	Facilitator/Barrier: Support from family and social networks plays a role in maintenance of health behaviors	Provide structured opportunities for peer connection and accountability by integrating peer-led discussions and collaborative activities
Cancer as a Catalyst for Behavior Change	Facilitator: Cancer diagnosis increases motivation to improve health behaviors	Leverage heightened motivation by offering timely dietary and physical activity guidance

## Data Availability

Data are available from the corresponding author upon reasonable request. The data are not publicly available due to privacy and ethical considerations, as the qualitative nature of the data, participant lived experiences, and small sample size could compromise participant anonymity.

## Supplementary Materials

The following supporting information can be downloaded at: https://www.mdpi.com/article/10.3390/nu18172896/s1, File S1: Interview guide and sample interview questions.

## References

[1] Keegan T.H.M., Abrahão R., Chubak J., Sauder C.A.M., Ruddy K.J., Quesenberry C., Li Q., Haupt E.C., Laurent C.A., Brunson A.M. (2025). Incidence of chronic medical conditions among survivors of adolescent and young adult cancer compared to a population without cancer. Cancer.

[2] Chao C., Bhatia S., Xu L., Cannavale K.L., Wong F.L., Huang P.S., Cooper R., Armenian S.H. (2020). Chronic Comorbidities Among Survivors of Adolescent and Young Adult Cancer. J. Clin. Oncol..

[3] Pekmezi D.W., Demark-Wahnefried W. (2011). Updated evidence in support of diet and exercise interventions in cancer survivors. Acta Oncol..

[4] Oeffinger K.C. (2008). Are survivors of acute lymphoblastic leukemia (ALL) at increased risk of cardiovascular disease?. Pediatr. Blood Cancer.

[5] Rabin C. (2017). Barriers to Increasing Physical Activity Among Young Adult Cancer Survivors. J. Adolesc. Young Adult Oncol..

[6] Powell-Wiley T.M., Poirier P., Burke L.E., Després J.-P., Gordon-Larsen P., Lavie C.J., Lear S.A., Ndumele C.E., Neeland I.J., Sanders P. (2021). Obesity and Cardiovascular Disease: A Scientific Statement From the American Heart Association. Circulation.

[7] Rock C.L., Thomson C.A., Sullivan K.R., Howe C.L., Kushi L.H., Caan B.J., Neuhouser M.L., Bandera E.V., Wang Y., Robien K. (2022). American Cancer Society nutrition and physical activity guideline for cancer survivors. CA Cancer J. Clin..

[8] Keats M.R., Culos-Reed S.N., Courneya K.S., McBride M. (2006). An examination of physical activity behaviors in a sample of adolescent cancer survivors. J. Pediatr. Oncol. Nurs..

[9] Kenkhuis M.F., Vlooswijk C., Janssen S.H.M., Kaal S.E.J., Tromp J.M., Bos M.E.M.M., van der Hulle T., Lalisang R.I., Nuver J., Bijlsma R.M. (2025). Associations of adherence to the World Cancer Research Fund lifestyle recommendations with health-related quality of life and fatigue in Adolescent and Young Adult (AYA) cancer survivors: Results from the SURVAYA study. J. Cancer Surviv..

[10] World Health Organization (2026). Healthy Diet.

[11] Forbes C., Tanner S., Engstrom T., Lee W.R., Patel D., Walker R., Bradford N., Pole J.D. (2024). Patient Reported Fatigue Among Adolescent and Young Adult Cancer Patients Compared to Non-Cancer Patients: A Systematic Review and Meta-Analysis. J. Adolesc. Young Adult Oncol..

[12] Geue K., Sender A., Schmidt R., Richter D., Hinz A., Schulte T., Brähler E., Stöbel-Richter Y. (2014). Gender-specific quality of life after cancer in young adulthood: A comparison with the general population. Qual. Life Res..

[13] Fox R.S., Armstrong G.E., Gaumond J.S., Vigoureux T.F.D., Miller C.H., Sanford S.D., Salsman J.M., Katsanis E., Badger T.A., Reed D.R. (2023). Social isolation and social connectedness among young adult cancer survivors: A systematic review. Cancer.

[14] Tanner S., Engstrom T., Forbes C., Patel D., Lee W.R., Walker R., Bradford N., Pole J.D. (2024). Physical function patient-reported outcomes among adolescent and young adult cancer survivors: A systematic review. Cancer Med..

[15] Valle C.G., Tate D.F., Mayer D.K., Allicock M., Cai J. (2013). A randomized trial of a Facebook-based physical activity intervention for young adult cancer survivors. J. Cancer Surviv..

[16] Johnson A.M., Baker K.S., Haviland M.J., Syrjala K.L., Abbey-Lambertz M., Chow E.J., Mendoza J.A. (2022). A Pilot Randomized Controlled Trial of a Fitbit- and Facebook-Based Physical Activity Intervention for Young Adult Cancer Survivors. J. Adolesc. Young Adult Oncol..

[17] Atkinson M., Murnane A., Goddard T., Pendergrast C., Rogers P., Manudhane R., Osborn M. (2021). A randomized controlled trial of a structured exercise intervention after the completion of acute cancer treatment in adolescents and young adults. Pediatr. Blood Cancer.

[18] Wurz A., Price J., Brunet J. (2021). Understanding adolescents’ and young adults’ self-perceptions after cancer treatment in the context of a two-arm, mixed-methods pilot randomized controlled physical activity trial. Support. Care Cancer.

[19] Quidde J., von Grundherr J., Koch B., Bokemeyer C., Escherich G., Valentini L., Buchholz D., Schilling G., Stein A. (2016). Improved nutrition in adolescents and young adults after childhood cancer-INAYA study. BMC Cancer.

[20] Grundherr J.V., Elmers S., Samland L., Mühlberg R., Dwinger S., Baumann J., Koch B., Wolters F., Hail L.A., Escherich G. (2026). Dietary intervention among young cancer survivors within the CARE for CAYA program. J. Cancer Surviv..

[21] Casillas J.N., Schwartz L.F., Crespi C.M., Ganz P.A., Kahn K.L., Stuber M.L., Bastani R., Alquaddomi F., Estrin D.L. (2019). The use of mobile technology and peer navigation to promote adolescent and young adult (AYA) cancer survivorship care: Results of a randomized controlled trial. J. Cancer Surviv..

[22] Schwartz L.A., Daniel L.C., Henry-Moss D., Bonafide C.P., Li Y., Psihogios A.M., Butler E.S., Szalda D., Ver Hoeve E.S., Hobbie W.L. (2020). Feasibility and acceptability of a pilot tailored text messaging intervention for adolescents and young adults completing cancer treatment. Psychooncology.

[23] Price J., Brunet J. (2022). Understanding rural-living young adult cancer survivors’ motivation during a telehealth behavior change intervention within a single-arm feasibility trial. Health Inform. J..

[24] DeNysschen C.A., Panek-Shirley L.M., Zimmerman B. (2021). Exercise with Nutrition Education to Improve Quality of Life of Adolescent and Young Adult Cancer Survivors: A Pilot Study. J. Adolesc. Young Adult Oncol..

[25] Vasilopoulou M., Asimakopoulou Z., Velissari J., Vicha A., Rizogianni M., Pusa S., Stöven S., Ficarra S., Bianco A., Jiménez-Pavón D. (2024). Interventions about physical activity and diet and their impact on adolescent and young adult cancer survivors: A Prisma systematic review. Support Care Cancer.

[26] Coffman E.M., Choi S.M., Smitherman A.B., Willis E.A., Martin S.L., Tate D.F., Valle C.G. (2025). Behavior change techniques in physical activity and dietary interventions among adolescent and young adult cancer survivors: A systematic review and meta-analysis of randomized controlled trials. J. Cancer Surviv..

[27] Hoover R.L., Xu J., Conklin J.L., Nichols H.B., Smitherman A., Valle C.G., Schwartz T., Mayer D.K., Hirschey R. (2024). Physical Activity Intervention Characteristics and Effects on Behavioral and Health-Related Outcomes Among Adolescents and Young Adults Living with and Beyond Cancer: A Systematic Review. J. Adolesc. Young Adult Oncol..

[28] Carmichael C.L., Reis H.T., Duberstein P.R. (2015). In your 20s it’s quantity, in your 30s it’s quality: The prognostic value of social activity across 30 years of adulthood. Psychol. Aging.

[29] Hopwood C.J., Donnellan M.B., Blonigen D.M., Krueger R.F., McGue M., Iacono W.G., Burt S.A. (2011). Genetic and environmental influences on personality trait stability and growth during the transition to adulthood: A three-wave longitudinal study. J. Pers. Soc. Psychol..

[30] Skiba M.B., McElfresh J.J., Howe C.L., Crane T.E., Kopp L.M., Jacobs E.T., Thomson C.A. (2020). Dietary Interventions for Adult Survivors of Adolescent and Young Adult Cancers: A Systematic Review and Narrative Synthesis. J. Adolesc. Young Adult Oncol..

[31] Crowder S.L., Hu B., Hoogland A.I., Gudenkauf L.M., Li X., Rodriguez Y., Irizarry-Arroyo N.E., Oswald L.B., Gonzalez B.D., Small B.J. (2025). Diet and physical activity intervention preferences for young adult cancer survivors. J. Cancer Surviv..

[32] Harris P.A., Taylor R., Minor B.L., Elliott V., Fernandez M., O’Neal L., McLeod L., Delacqua G., Delacqua F., Kirby J. (2019). The REDCap consortium: Building an international community of software partners. J. Biomed. Inform..

[33] Harris P.A., Taylor R., Thielke R., Payne J., Gonzalez N., Conde J.G. (2009). Research electronic data capture (REDCap)—A metadata-driven methodology and workflow process for providing translational research informatics support. J. Biomed. Inform..

[34] Guest G., MacQueen K.M., Namey E.E. (2011). Applied Thematic Analysis.

[35] McHugh M.L. (2012). Interrater reliability: The kappa statistic. Biochem. Med..

[36] Lumivero (2020). NVivo (Version 12.0) [Computer Software]. https://lumivero.com/products/nvivo/.

[37] Bandura A. (2001). Social cognitive theory: An agentic perspective. Annu. Rev. Psychol..

[38] (2013). SAS Software.

[39] Coa K.I., Smith K.C., Klassen A.C., Caulfield L.E., Helzlsouer K., Peairs K., Shockney L. (2015). Capitalizing on the “teachable moment” to promote healthy dietary changes among cancer survivors: The perspectives of health care providers. Support. Care Cancer.

[40] Lau N., Steineck A., Walsh C., Fladeboe K.M., Yi-Frazier J.P., Rosenberg A.R., Barton K. (2024). Social support resources in adolescents and young adults with advanced cancer: A qualitative analysis. BMC Palliat. Care.

[41] Wickramasinghe B., Fern L.A., Taylor R.M., Feltbower R.G. (2025). Longitudinal Cohort Study of the Relationship Between Illness Perception, Perceived Social Support, and Psychosocial Quality of Life in Adolescents and Young Adults Newly Diagnosed with Cancer: Outcomes from a BRIGHTLIGHT Study. Cancers.

[42] Buro A.W., Sauls R., Stern M., Carson T.L. (2023). A qualitative study of stress experiences, health behaviors, and intervention preferences in young adult cancer survivors. Support. Care Cancer.

[43] Wu Y.P., Yi J., McClellan J., Kim J., Tian T., Grahmann B., Kirchhoff A.C., Holton A., Wright J. (2015). Barriers and Facilitators of Healthy Diet and Exercise Among Adolescent and Young Adult Cancer Survivors: Implications for Behavioral Interventions. J. Adolesc. Young Adult Oncol..

[44] Sleeman S.H.E., Reuvers M.J.P., van der Veldt M.H., Manten-Horst E., Husson O. (2025). ‘What Really Goes on in My Cancer Bubble, They Cannot Understand’: Social Functioning Among Adolescent and Young Adult (AYA) Cancer Patients. Curr. Oncol..

[45] Nowe E., Friedrich M., Leuteritz K., Sender A., Stöbel-Richter Y., Schulte T., Hinz A., Geue K. (2019). Cancer-Related Fatigue and Associated Factors in Young Adult Cancer Patients. J. Adolesc. Young Adult Oncol..

[46] Murnane A., Kiss N., Fraser S.F., Lewin J. (2021). Health-related quality of life, fatigue and health behaviours in Australian adolescent and young adult cancer survivors. Pediatr. Blood Cancer.

[47] Angoumis K., Padilla C.S., Kouwenhoven M.C.M., Bijlsma R.M., Kaal S.E.J., Tromp J.M., Bos M., van der Hulle T., Broen M.P.G., Nuver J. (2025). Adverse health outcomes and health-related quality of life (HRQoL) among long-term adolescent and young adult (AYA) brain tumour survivors: Results from the population-based SURVAYA study. Support. Care Cancer.

[48] Braun I., Friedrich M., Morgenstern L., Sender A., Geue K., Mehnert-Theuerkauf A., Leuteritz K. (2023). Changes, challenges and support in work, education and finances of adolescent and young adult (AYA) cancer survivors: A qualitative study. Eur. J. Oncol. Nurs..

[49] Ghazal L.V., Gormley M., Merriman J.D., Santacroce S.J. (2021). Financial Toxicity in Adolescents and Young Adults With Cancer: A Concept Analysis. Cancer Nurs..

[50] Kaddas H.K., Pannier S.T., Mann K., Waters A.R., Salmon S., Tsukamoto T., Warner E.L., Fowler B., Lewis M.A., Fair D.B. (2019). Age-Related Differences in Financial Toxicity and Unmet Resource Needs Among Adolescent and Young Adult Cancer Patients. J. Adolesc. Young Adult Oncol..

[51] Kirchhoff A., Jones S. (2021). Financial Toxicity in Adolescent and Young Adult Cancer Survivors: Proposed Directions for Future Research. JNCI J. Natl. Cancer Inst..

[52] Parsons H.M., Harlan L.C., Lynch C.F., Hamilton A.S., Wu X.C., Kato I., Schwartz S.M., Smith A.W., Keel G., Keegan T.H. (2012). Impact of cancer on work and education among adolescent and young adult cancer survivors. J. Clin. Oncol..

[53] Li X., Hathaway C.A., Small B.J., Tometich D.B., Gudenkauf L.M., Hoogland A.I., Fox R.S., Victorson D.E., Salsman J.M., Gonzalez B.D. (2024). Social isolation, depression, and anxiety among young adult cancer survivors: The mediating role of social connectedness. Cancer.

[54] Ander M., Thorsell Cederberg J., von Essen L., Hovén E. (2018). Exploration of psychological distress experienced by survivors of adolescent cancer reporting a need for psychological support. PLoS ONE.

[55] Murnane A., Kiss N., Lewin J., Fraser S.F., Ugalde A. (2025). Optimizing Support for Adolescent and Young Adult Cancer Survivors: Recommendations on Exercise, Nutrition, and Post-Treatment Care Needs from a Qualitative Study. J. Adolesc. Young Adult Oncol..

[56] Berkman A.M., Betts A.C., Beauchemin M., Parsons S.K., Freyer D.R., Roth M.E. (2024). Survivorship after adolescent and young adult cancer: Models of care, disparities, and opportunities. J. Natl. Cancer Inst..

