# Supplementary material for: Perceived Facilitators and Barriers Inform Young Adult Cancer Survivors’ Preferences for Physical Activity and Diet Interventions: A Qualitative Study
Source: Nutrients. 2026 Sep 3;18(17):2896. doi: 10.3390/nu18172896 (PMC13567726; doi:10.3390/nu18172896)
Supplement: Supplementary file 1 [file nutrients-18-02896-s001.zip › nutrients-4490275-supplementary.pdf]

Supplemental File S1.

Interview guide and sample interview questions

Introduction

2 minutes of free discussion and rapport building. Take notes if the participant mentions a topic that will be addressed after the recording begins.

Let me tell you a little more about the study before we jump into the interview. Again, thank you for agreeing to participate in this study. To review, the purpose of this study is to examine factors related to eating and physical activity, barriers and facilitators, and intervention format preferences in young adult cancer survivorship. We also want to use these interviews to help us develop a diet and physical activity intervention preferable to young adult cancer survivors to improve quality of life.

We will spend about 60 minutes discussing a range of topics. You are the expert in your medical problems, your life, and which aspects are most important to you. This is what we would like to better understand. Please know that there are no right or wrong answers. Just do your best to respond with your honest thoughts and feelings.

We will be recording the interview so we can stay focused on the conversation we are having instead of being distracted by taking notes. We will use this recording for research purposes only. Your personal information and everything we talk about will remain confidential. Some quotations and major themes we discuss will be used in research but reported anonymously, which means we will say things like "one participant said," or "a few people said." None of our conversation will be part of your medical record or shared with your medical providers. We want you to be able to speak freely. If you are uncomfortable at any point and would like to stop the recording, please let me know.

I will guide the discussion so we can be sure to cover a few key topics, but this is meant to be relatively conversational so do not be concerned if we stray off topic. I will keep track of the time and what we have covered, and if we need to move on to the next topic, then I will let you know. If any part of the discussion causes you concern about your health or raises questions for you, we can provide a number for our social worker or you are welcome to contact your medical provider after the interview. If you need to break at any point or need to stop the interview, just let me know. You can also choose to skip any question, and whether you skip a question or choose to stop, it will not affect your care at Moffitt.

To review, the purpose of this interview is to learn about preferences and barriers to diet and physical activity.

Do you have any questions before we begin? [Answer questions]

Let's begin the interview. I will start the recording now. [Start recording] This is participant [specify ID number], interviewed by [name of interviewer] on [date] at [time].

<u>Quality of Life and Health Behaviors</u>

Because we are working with your doctor on this study, we know a little bit about your cancer history. What we do not know is what it has been like for you to have cancer and receive these treatments.

- Tell me about how you were first diagnosed with cancer. What was that like for you?
    - What were your biggest concerns?
    - How did your life change?
    - What kind of symptoms and side-effects did you have that impacted your ability to eat or participate in physical activity?
    - How did [insert specific symptoms] impact your life?
    - What parts of your life were most improved?
    - What parts of your life were made worse?

<u>Survivorship Guidelines- Nutrition and Physical Activity</u>

- Tell me about any sociocultural factors related to diet and physical activity.
    - How do you decide to eat what you eat- what food do you like/dislike? (probe preference/barriers)
    - What types of physical activity do you like/dislike? (probe preferences/barriers)
    - What kind of changes did you make to your health habits (diet, exercise, sleep)?
    - What kind of impact did these changes to your habits have on your health (symptoms, well-being, overall health)?

Physical Activity:

- What has your physical activity been like since treatment completion?

- The American Cancer Society created specific guidelines for physical activity. Are you familiar with them?
    - If yes, probe for who told patient about them -- oncologist, physical therapist, dietitian, exercise specialist, primary doc, etc.

- The American Cancer Society exercise guidelines recommend 150 min a week of exercise including strength training for 2 days a week. To what extent are you meeting the exercise guidelines?
    - If NOT meeting exercise guidelines, what is holding you back from exercising?
    - If YES, what types of exercise do you do?
    - If MEETING guidelines, how many minutes per week do you spend exercising

- Overall, how important is physical activity to you?

Nutrition:

- How would you compare your current diet to your diet before treatment?

- The American Cancer Society also created guidelines for nutrition? Are you familiar with them?
    - If yes, probe for who told oncologist, physical therapist, dietitian, exercise specialist, primary doc, etc.

- The American Cancer Society guidelines recommend limiting the consumption of high-calorie foods and beverages and achieving a diet high in fruits, vegetables, and whole grains. Do you feel you are meeting these nutrition guidelines?
    - If YES, can you share what you commonly eat with me?
    - If NOT meeting nutrition guidelines, what is holding you back?

- Overall, how important is nutrition to you?

Dietary Counseling and Physical Activity Programming

As I mentioned in the introduction, we are planning to use these interviews to develop a diet and physical activity intervention to improve quality of life in young adult cancer survivors.

a. What are your first impressions about a program like that?
b. Do you think that a program like that could be helpful? In what ways?
c. Do you think a program like that could be unhelpful? In what ways?

- Would you be interested in receiving nutrition counseling and physical activity sessions?
    - If YES,
    - Would you prefer these sessions to be group-based or individual?
    - Would you prefer all men or all women or mixed genders?
    - Would you prefer online or in-person sessions
        - online probe for modality: app, text messages, email, ZOOM, etc.
        - in-person probe as to where: hospital, park, local gym, etc.
    - Timing of sessions
        - during treatment, immediately following, 6 months after, etc.
    - How often would you like the sessions?
        - Times per week and minutes per week ex. 3 x week for 30 minutes
    - Preferred time-period: morning, afternoon, evening
    - Would you be interested in setting goals for improving health habits (like exercise, diet, relaxation/sleep)?
    - Would you be open to talking about your values (what is important to you), and how you might live a life consistent with your values?
    - What types of topics would you like the diet and physical activity sessions to include?
        - Examples: virtual teaching kitchen cooking classes, fitness classes, reading nutrition labels, eating healthy on a budget, etc.

- From whom would you be interested in receiving a diet and physical activity intervention from? – exercise specialist, registered dietitian, nurse, etc.

- What types of obstacles do you have that would prevent for from participating in a diet and physical activity intervention?

- What do you anticipate a diet and physical activity intervention to impact your overall well-being?

- Do you have any specific intervention preferences as a function of your:
    - culture
    - gender/biological sex
    - cancer stage/type/treatment?

Technology Questions

- What did you like about tracking your physical activity on the Fitbit?
    - Dislike?

- What did you like about completing the 3-day food recalls and digital diet diaries?
    - Dislike?

- Do you have any additional feedback on the platforms (digital aspects, Fitbit ease/acceptability)?

<u>Assess for Unmet Needs</u>

Is there anything else that you would like to add? Is there any important issue in your life that we haven't talked about that you would like to share with me?

1. How have you felt about taking part in this interview? Is there anything you would like to ask me about the interview?

“If anything we discussed bothers you or starts to cause you worry, please reach out to us and our social worker will contact you. If you think you need to speak with someone now, I can provide that contact information for you at this time. Thank you for your time.”

INTERVIEWER NOTES / REFLECTIONS ON INTERVIEW:
